# Validating automated screening for psychological distress by means of computer touchscreens for use in routine oncology practice

**DOI:** 10.1054/bjoc.2001.2182

**Published:** 2001-12

**Authors:** A Cull, A Gould, A House, A Smith, V Strong, G Velikova, P Wright, P Selby

**Affiliations:** Imperial Cancer Research Fund, Medical Oncology Unit, Western General Hospital, Edinburgh, UK; Information and Statistics Division, NHS in Scotland, Edinburgh, UK; Department of Liaison Psychiatry, University of Leeds, UK; Imperial Cancer Research Fund, Cancer Medicine Research Unit, St James's University Hospital, Leeds, UK

**Keywords:** screening, psychological distress, computer touchscreens, routine practice, oncology

## Abstract

The aim of the study was to confirm the validity of using touchscreen computers for screening for clinically significant levels of distress among cancer patients in routine oncology practice. The Hospital Anxiety and Depression Scale (HADS), EORTC Quality of Life questionnaire (QLQ-C30), Mental Health Inventory-MHI5 and a Concerns Checklist were administered via touchscreen computer to 172 chemotherapy out-patients, twice, 2–4 weeks apart. A standard psychiatric interview (Present State Examination – PSE) was conducted within a week of the second assessment. On interview, 23% of patients were identified as ‘cases’. Using the available data (questionnaires, sociodemographic details, self-reported past psychiatric history), the best screening strategy combined scores from MHI-5 and HADS from a single time-point with the following rules: if MHI-5 < 11 = non-case; if MHI-5 ≥ 11 then use HADS; then, if HADS ≥ 9 = ‘case’ (sensitivity 85%; specificity 71%; misclassification rate 26%; positive predictive value 47%). The computerized screening system enabled data to be collected, scored, collated and reported in real time to identify patients who warrant further clinical assessment. It offers the potential for improving ‘case’ detection in routine oncology practice while reducing the burden of questions put to ‘non-cases’. Further work is needed to develop optimal choice of screening questions for this purpose. © 2001 Cancer Research Campaign http://www.bjcancer.com


British Journal of Cancer (2001) 85(12), 1842–1849
© 2001 Cancer Research Campaign
doi: 10.1054/ bjoc.2001.2182, available online at http://www.idealibrary.com on IDEAL

http://www.bjcancer.com

### Validating automated screening for psychological distress by means of computer touchscreens for use in routine oncology practice

**A Cull[1], A Gould[2], A House[3], A Smith[4], V Strong[1], G Velikova[4], P Wright[4] and P Selby[4]**

[1]Imperial Cancer Research Fund, Medical Oncology Unit, Western General Hospital, Edinburgh; UK; [2]Information and Statistics Division, NHS in Scotland, Edinburgh, UK; [3]Department of Liaison Psychiatry, University of Leeds, UK; [4]Imperial Cancer Research Fund, Cancer Medicine Research Unit, St James's University Hospital, Leeds, UK

**Summary** The aim of the study was to confirm the validity of using touchscreen computers for screening for clinically significant levels of distress among cancer patients in routine oncology practice. The Hospital Anxiety and Depression Scale (HADS), EORTC Quality of Life questionnaire (QLQ-C30), Mental Health Inventory-MHI5 and a Concerns Checklist were administered via touchscreen computer to 172 chemotherapy out-patients, twice, 2–4 weeks apart. A standard psychiatric interview (Present State Examination – PSE) was conducted within a week of the second assessment. On interview, 23% of patients were identified as 'cases'. Using the available data (questionnaires, sociodemographic details, self-reported past psychiatric history), the best screening strategy combined scores from MHI-5 and HADS from a single time-point with the following rules: if MHI-5 < 11 = non-case; if MHI-5 ≥ 11 then use HADS; then, if HADS ≥ 9 = 'case' (sensitivity 85%; specificity 71%; misclassification rate 26%; positive predictive value 47%). The computerized screening system enabled data to be collected, scored, collated and reported in real time to identify patients who warrant further clinical assessment. It offers the potential for improving 'case' detection in routine oncology practice while reducing the burden of questions put to 'non-cases'. Further work is needed to develop optimal choice of screening questions for this purpose. © 2001 Cancer Research Campaign http://www.bjcancer.com

**Keywords**: screening; psychological distress; computer touchscreens; routine practice; oncology

Systematic studies have shown that major depression, anxiety states and adjustment disorders affect a significant proportion of cancer patients to a degree that warrants intervention (Pinder et al, 1993; Aapro and Cull, 1999). These problems often go undetected and untreated in routine oncology practice (Maguire, 1985; Cull et al, 1995; Berard et al, 1998; Newell et al, 1998; Fallowfield et al, 2001). Psychological comorbidity is associated with an increased symptom burden, greater disability, poorer quality of life, reduced compliance with medical treatment and a poorer medical outcome for patient (Ramirez and House, 1997). It also constitutes an increased burden for their carers. There is now ample evidence of the efficacy of a range of psychological interventions (Meyer and Mark, 1995) in relieving cancer patients' distress. In most health care systems the resources to provide these interventions are limited and need to be targeted appropriately.

Training in communication and counselling skills has been increasingly available to oncology staff in recent years (Maguire et al, 1996; Fallowfield et al, 1998). This should improve early recognition of, and appropriate response to, patients' concerns and difficulties and may prevent problems from escalating. Even so, an initial screening strategy based on patient self-report questionnaire data may help staff in busy clinics to concentrate their efforts on those most in need and to develop a rational basis for referral of patients for specialist intervention. The administrative burden of administering and scoring questionnaires hampers screening in routine oncology practice. Electronic methods of data capture offer the means of developing a practical and efficient screening strategy for routine use.

Comparing questionnaire administration by computer touchscreen with paper forms subsequently scanned by an optical reading system, we found the touchscreen system was well accepted by oncology in-patients, quicker to use and provided better-quality data. Agreement of scores between electronic and paper forms and the test–retest reliability of the touchscreen data was generally good (Velikova et al, 1999). In the present study the automated system was applied in the outpatient setting to identify patients whose questionnaire scores suggested clinically significant levels of distress which would warrant further assessment in the clinic.

The optimal choice of screening tool(s) for this purpose remains controversial. We were influenced in our selection of measures by those already in use, e.g. for clinical trials, as well as the need for economy in the total number of questions asked. The Hospital Anxiety and Depression Scale (HADS) (Zigmond and Snaith, 1983) has been so widely used to assess psychological distress in cancer patients in Europe that it appeared to be the first choice. However the optimal choice of cut-off score for 'case-identification' is controversial. Validation studies of the HADS against a standard psychiatric interview in homogeneous samples of breast cancer (Razavi et al, 1990; Hopwood et al, 1991a, b; Ramirez et al, 1995; Hall et al, 1999) or lymphoma (Razavi et al, 1992) patients have reported equivocal results about its screening performance. In a heterogeneous sample Ibbotson et al (1994) recommended different cut-off scores for optimal case identification for patients with

*Received 21 March 2001*
*Revised 2 August 2001*
*Accepted 18 September 2001*

*Correspondence to:* A Cull different disease or treatment characteristics, i.e. disease-free, stable or progressive disease; on or off treatment. In our study, patients could not be asked to supply this information on their arrival at clinic nor was it reliably accessible electronically from patient records. We therefore sought alternative means of improving the screening performance of the HADS. We considered that sequential HADS scores might provide more effective screening in a setting where patients return regularly to the oncology clinic.

We also included possible alternatives or adjuncts to the HADS. The 5-item Mental Health Inventory (Berwick et al, 1991), a component of the SF-36 Health Survey (Ware, 1993), has been validated as a screening tool against the Diagnostic Interview Schedule. Potentially it offered a briefer alternative to the HADS. In oncology clinics the EORTC QLQ-C30 (Aaronson et al, 1993) has been shown to facilitate doctor–patient communication about both physical and psychological functioning (Detmar and Aaronson, 1998). It includes a 4-item emotional functioning (EF) scale which has not yet been evaluated as a screening tool. The nature and number of cancer patients' concerns as assessed by a brief checklist have been shown to be a useful marker for psychiatric morbidity (Harrison et al, 1994). The checklist provides a helpful starting point for remedial intervention by identifying concerns contributing to patients' distress. The screening performance of any of these measures could potentially be improved by including information about known risk factors for psychological morbidity, such as gender or past psychiatric history (Robertson and Katona, 1997). The aim of this study was to assess the validity of computer-administered screening questionnaires against a standard psychiatric interview for detecting clinically significant levels of psychological distress in a mixed group of cancer patients in routine oncology practice. Using the touchscreen system as the screening platform, the specific objectives were:

1. To determine the prevalence of clinically significant psych logical morbidity as assessed by a standard clinical interview.
2. To compare the screening performance of the individual measures selected for use in this study.
3. To assess whether the identification of 'cases' is improved by using 2 sequential questionnaire assessments relative to data from a single time point.
4. To determine the optimal selection of measures (singly or in combination) for 'case' identification in this setting.

### PATIENTS AND METHODS

### Patients

Participants were day-patients attending for chemotherapy in 2 cancer centres in the UK. To be eligible for this study patients had to be able to read English (to complete the questionnaires on the touchscreen), to have 2 appointments at the hospital in a 6-week period (after giving their consent) and to be available for interview (at home or in hospital) within a week of the second questionnaire assessment.

### Instruments

*HADS* (Zigmond and Snaith, 1983). This instrument consists of 27-item subscales to measure anxiety and depression. Patients respond by endorsing the response which best describes how they have been feeling in the past week. Items are scored (0–3) and summed to give subscale scores (0–21) for anxiety and depression, and a total score (0–42). Higher scores denote greater distress.

*Mental Health Inventory – MHI-5* – UK version (Ware, 1993). This consists of questions about anxiety ($n$ = 1), depression ($n$ = 1), loss of behavioural/emotional control ($n$ = 1) and psychological wellbeing ($n$ = 2). Patients respond in terms of how much of the time in the past 4 weeks they have been feeling like this from 1 (all the time) to 6 (none of the time). Berwick's original scoring method (1991) was used to adjust for the direction of the items. A higher score on the summary scale score (range 5–30) indicates more distress.

*EORTC QLQ-C30, Emotional functioning scale – EF* (Aaronson et al, 1993). This 4-item scale contains items covering tension, worry, irritability and depression experienced during the past week. Responses are given on a 4-point scale from 1 (not at all) to 4 (very much). The summary scale is linearly transformed to a 0–100 scale where a higher score means better functioning.

*Concerns Checklist* (Harrison et al, 1994). This 14-item checklist refers to 13 cancer-related concerns (current illness, physical symptoms, treatment, feeling different, feeling upset, inability to do things, future, job, finances, relationship with partner, relationship with others, sexuality, amount of support) and an open 'other' category. Patients rate how worrying they appraised each concern to have been over 'the last few weeks' from 0 (not at all) to 4 (extremely). Ratings for each item were summed to give an aggregate score (0–56). A higher score represents more worry.

*Past Psychiatric History – PH*. Patients were asked to respond yes/no to the following 2 questions (House, personal communication): Have you ever been treated for nervous or emotional problems such as anxiety or depression in the past at any time? – PH1; Have you ever had an admission to hospital for nervous problems? – PH2.

*Present State Examination – PSE* (Wing et al, 1974). A short form of this well validated and widely used psychiatric interview schedule was used to elicit and rate psychiatric symptoms experienced over the preceding month. Ratings were analysed using the Catego computer program to detect 'cases' from the Index of Definition (ID). This comprises a number of levels of certainty that a psychiatric disorder is present from no symptoms at all (ID1) through threshold disorders (ID5) to definite cases (ID6–8). An individual who rates ID $\geq$ 5 can be given a diagnosis using ICD-10 with the addition of a few extra questions to the PSE interview. These questions, which do not affect the ID ratings, were included in our interviews but are not separately reported here.

### Procedure

Eligible patients were given written information and invited to take part in the study by one of the research team in each centre (VS, PW). Diagnostic and treatment data were extracted from the patients' casenotes. Patients were asked to give the following sociodemographic information: age, sex, marital status, living arrangements and postcode. The Social Deprivation Index (SDI) was derived by translating patients' postcodes into Carstairs scores (Carstairs and Morris, 1991) which were expressed as quintiles from 1 = most affluent to 5 = most socially deprived. General practitioners were informed of their patients' participation in the study. Patients were shown how to use the touchscreen monitor and asked to complete the computer-administered questionnaires on 2 separate clinic visits, a minimum of 2 and a maximum of 4 weeks

*© 2001 Cancer Research Campaign*

apart. Patients were interviewed by an interviewer trained in the use of the PSE (VS, PW), either at the clinic after the second questionnaire assessment or in their own homes in the ensuing week. The interviewers, who were blind to the touchscreen scores, scored patients' responses on the standard interview rating schedule. These data were entered into the Catego computer program. All interviews were recorded on audiotape (subject to the patient giving written informed consent). At the end of the study interviewers re-rated audiotapes of a randomly selected 10% of their own interviews (without reference to their original ratings). Interviewers' re-ratings were concordant with their original ratings on 96% of the interviews sampled. A further 10% of interviews were exchanged between the interviewers who rated the audiotapes blind to the original ratings. Inter-rater reliability was satisfactory with 88% concordance in case identification between the 2 interviewers. An experienced PSE trainer (AH) independently rated the interviews in question and discrepancies were resolved by consensus.

### Statistical methods

The statistical analysis was carried out using the S-PLUS system (1997). For comparison of proportions the $\chi^2$ test was used (Armitage, 1971). Logistic regression (McCullagh and Nelder, 1989) was used to build models to predict 'caseness'. A cross-validated variable selection (Harrell et al, 1998, 2000) was used to find the most predictive of the variables and to provide robust estimates of the $R^2$ statistic. These 'corrected' values of $R^2$ provide a less over-optimistic prediction of the performance of the model when applied to data which were not used to build it. Receiver operating characteristic (ROC) curves (Murphy et al, 1987) were used to represent the ability of the measures to discriminate between 'cases' and 'not cases'. The area under the ROC curve, or AUC provides a summary measure of the performance of a screening decision rule across the range of possible cut-off points. The higher the AUC, the better the predictive ability of the rule. The cross-validated variable selection method referred to above also provided robust estimates of the areas under the ROC curves. These estimates are referred to as 'corrected' in the text. The screening performance of the selected instruments was assessed by reference to their sensitivity and specificity. Sensitivity refers to the proportion of correctly identified 'cases' (true case/true positives + false negatives). Specificity refers to the proportion of correctly identified non-cases (true negatives/true negatives + false positives). Hence the false positive rate is 1 – specificity. The positive predictive value (PPV) was calculated to give the probability of being a 'case' at a given cut-off point (true positives/true positives + false positives). The misclassification rate (MR) refers to the number of patients misclassified (false negatives + false positives/ total no. of patients) by the screening instrument in question. A decision tree was used to confirm the optimal screening strategy (Venables and Riplay, 1997). Cross-validation methods were also applied to avoid over-fitting of the data.

### RESULTS

Of 298 patients approached 40 (13%) refused to take part in the study and 15 (5%) withdrew after the first assessment (response rate 82%). A further 36 (12%) patients had subsequently to be excluded from the analysis for clinical reasons when a change in their health status or treatment plan meant that the assessments could not be completed in the time-frame required by our protocol. Of 207 patients who underwent the PSE interview, 35 patients were withdrawn from the analysis after technical problems resulted in the loss of one or both sets of questionnaire data. A complete dataset, of 2 sets of questionnaire data from touchscreen administration (collected 2–4 weeks apart) and a PSE interview, was available for 172 patients in the 2 centres.

The sample consisted of 66 (38%) men and 106 (62%) women in the age range 21–81 years (median = 57). The majority were married (69%); 16% lived alone. The SDI showed the following distribution: 1 (most affluent) = 24%; 2 = 19%; 3 = 19%; 4 = 20%; 5 (most deprived) = 14%. Postcodes were unknown for 4% of the sample. The majority (99%) of patients were white. Patients with colorectal ($n$ = 49), breast ($n$ = 37) or ovarian ($n$ = 34) cancer or lymphoma ($n$ = 19) accounted for 81% of the sample. The remaining 33 patients were heterogeneous with respect to their cancer diagnosis. Responses to the questions about psychiatric history showed that 22% of the sample had had some history of treatment for 'nervous or emotional problems' in the past (PH1) and for 4% this had warranted hospital treatment (PH2).

#### *1. Prevalence of clinically significant psychiatric morbidity*

From 172 PSE interviews 16 patients (9%) were identified as 'definite cases' (Catego ID scores 6–8) and a further 24 (14%) as 'threshold disorders' (Catego ID score = 5), i.e. a total of 40 (23%) patients whose level of distress warranted some clinical intervention. In the analyses which follow patients with Catego ID scores ≥ 5 are designated 'cases'. The Catego ID scores of the 35 patients who had had to be excluded from the main study analysis because of incomplete touchscreen data were not significantly different from the Catego scores of these patients with full data ($\chi^2$ = 3.78, df = 6, $P$ = 0.71). Table 1 shows (a) the distribution of sociodemographic and past psychiatric history characteristics in the sample, (b) the percentage of patients with each of these characteristics who were identified as 'cases' and (c) the distribution of these characteristics among the 40 patients identified as 'cases'. The only variable showing a significant association with Catego 'caseness' on univariate analysis was past psychiatric history (PH1). Patients who had previously been treated for nervous or emotional problems were twice as likely to be identified as 'cases' on clinical interview as patients with no such history (38% vs 19%; $\chi^2$ = 5.62, df = 1, $P$ = 0.02).

The following variables were entered into a logistic regression analysis to predict 'caseness': sex, age, marital status, living status, SDI and the responses to the 2 questions about past psychiatric history (PH1, PH2). Age and SDI were treated as continuous variables in the regression analysis. Cancer diagnosis was not included in the model. Younger age (odds ratio 0.96, (95% confidence interval: 0.93–0.99) for each successive year of age) and past psychiatric history, as defined by an affirmative response to PH1, (odds ratio 2.47, 95% CI: 1.00–6.11) were significant in the prediction of 'caseness'.

#### *2. Comparison of the performance of the screening questionnaires used in the study*

There were no significant differences between scores at the first and second administration for any of the measures used. Data from the second administration i.e. temporally closest to the interview, were examined first. The median scores (and ranges) from the second assessment were as follows: HADS 9.0 (0–31); MHI5 10.0 (5–22); EORTC QLQ-C30 75.0 (16.7–100); Concerns 8.0 (0–34).

 *© 2001 Cancer Research Campaign*

**Table 1** Sociodemographic and clinical characteristics by 'caseness' on PSE interview

| Variable | No. of patients | % of patients with this characteristic who have Catego ID score ≥ 5 | % of 'cases' i.e. Catego ID score ≥ 5 who have this characteristic (i.e. of $n$ = 40) |
|---|---|---|---|
| Gender | | | |
| men | 66 | 17 | 28 |
| women | 106 | 27 | 72 |
| Age | | | |
| < 50 | 50 | 30 | 38 |
| ≥ 50 | 122 | 21 | 62 |
| Marital status | | | |
| single | 20 | 25 | 13 |
| married | 119 | 22 | 65 |
| separated/widowed/divorced | 33 | 27 | 22 |
| Living status | | | |
| alone | 28 | 18 | 13 |
| with others | 144 | 24 | 87 |
| SDI[a] | | | |
| 1/2 | 74 | 23 | 43 |
| 3 | 32 | 22 | 17 |
| 4/5 | 59 | 24 | 35 |
| Diagnosis | | | |
| breast | 37 | 22 | 20 |
| ovarian | 34 | 32 | 28 |
| colorectal | 49 | 18 | 22 |
| lymphoma | 19 | 11 | 5 |
| other | 33 | 30 | 25 |
| Past psych history (PH1) | | | |
| Yes | 37 | 38 | 35 |
| No | 135 | 19 | 65 |
| Past psych admission (PH2) | | | |
| Yes | 7 | 29 | 5 |
| No | 165 | 23 | 95 |

[a]7 patients had no SDI recorded; 2 of them were cases.

**Table 2** Correlation between questionnaire scores (2nd assessment) and, for each questionnaire, with Catego scores (Pearson's r)

| | HADS.2 | MH15.2 | EF.2 | Concerns.2 |
|---|---|---|---|---|
| HADS.2 | – | 0.77 | – 0.71 | 0.74 |
| MH1–5.2 | – | – | – 0.61 | 0.63 |
| EF.2 | – | – | – | – 0.63 |
| Concerns.2 | – | – | – | – |
| Catego | 0.57 | 0.55 | – 0.45 | 0.49 |

Scores on the 4 questionnaire measures were highly inter-correlated with each other (Table 2) and in each case showed a significant ($P < 0.001$) linear relationship with Catego ID scores.

Logistic regression was used to assess the performance of the 4 individual questionnaires in predicting 'caseness'. Each questionnaire was tested separately in a model with the same sociodemographic and psychiatric history variables as before. None of the sociodemographic or psychiatric history variables was significant in a model where scores from at least one of the questionnaires were included. Taken singly, each of the questionnaires was significant ($P < 0.001$) in these models. HADS and MHI-5 scores were the best single predictors of 'caseness' as judged by the highest $R^2$ and area under the ROC curve (AUC).

### *3. Comparing data from 2 sequential assessments with data from a single time point*

A logistic regression model was built to predict 'caseness' using the sociodemographic data as before and all the scores from the second assessment (Model 1). Then questionnaire data from the first assessment were added (Model 2). The models were compared. Model 2 fits the data significantly better than Model 1 ($\chi^2 = 10.4$, df = 4, $P = 0.03$) indicating that the inclusion of the questionnaire data from 2 time points did improve the prediction of 'caseness' when all the measures used were included in the analysis.

### *4. The optimal selection of measures (singly or in combination) for case-identification*

The most parsimonious sub-models of Models 1 and 2 were found using cross-validated stepwise elimination of the variables. The reduced models, Model 1a and 2a respectively, are specified in the footnote of Table 3. Both of these models fit the data as well as the full models and the corrected $R^2$ and AUC statistics are improved. This reflects the probable overfitting in the 'full' models 1 and 2. Models 1a and 2a are very similar in terms of their corrected $R^2$ and AUC. In both cases only the HADS and MHI5 are included. Models were generated using all other possible combinations of HADS and MHI5 scores. Combining data from two administrations of the HADS was significantly better than predicting 'caseness' from a single HADS ($\chi^2 = 4.04$, df = 1, $P = 0.04$). Of all the

*© 2001 Cancer Research Campaign* 

**Table 3** Comparison of the performance of screening strategies: using HADS and MH15 singly and in optimal combination (Models 1a and 2a and decision trees)

| | Cutpoint[a] | Sensitivity (%) | Specificity (%) | Positive predictive value (%) | Misclassification rate (%) |
|---|---|---|---|---|---|
| HADS1 | 13 | 60 | 83 | 52 | 22 |
| | 15 | 48 | 89 | 57 | 21 |
| | 19 | 33 | 98 | 83 | 17 |
| MH15.1 | 10 | 85 | 52 | 35 | 40 |
| | 11 | 70 | 63 | 36 | 35 |
| HADS2 | 13 | 60 | 85 | 55 | 21 |
| | 15 | 50 | 91 | 63 | 19 |
| | 19 | 25 | 99 | 88 | 18 |
| MH15.2 | 9 | 85 | 54 | 36 | 39 |
| | 11 | 70 | 72 | 43 | 28 |
| Model 1a[b] | – 1.9 | 85 | 58 | 38 | 36 |
| | – 1.2 | 70 | 75 | 46 | 26 |
| Model 2a[c] | – 2.0 | 85 | 58 | 38 | 36 |
| | – 1.2 | 70 | 75 | 46 | 26 |
| Decision tree using HADS1 and MH15.1 | See Figure 2 | 85 | 71 | 47 | 26 |
| Decision tree applied to HADS2, MHI5.2 | As per Figure 2 | 73 | 70 | 43 | 29 |

[a]Classify as case if scoring higher than this value. [b]Model 1a: score = –5.06 + 0.13*HADS.2 + 0.20*MH15.2.
[c]Model 2a: score = –5.64 + 0.13*HADS1 + 0.24*MH15.2.

regression models generated, Model 2a fitted the data best by every criterion although the differences between the models were not statistically significant.

The performance of the screening strategies implied by these analyses was compared (Table 3). The screening performance of HADS was assessed with respect to cut-offs cited in the literature (Razavi et al, 1990; Ibbotson et al, 1994). For ease of comparison in Table 3 the screening performance of MHI5 and the 2 best regression models (1a and 2a) are described for sensitivity values of 85% and 70%. For HADS.2 to achieve a sensitivity of 85% the cutoff score had to be reduced to 7, resulting in a specificity of only 48%.

Linear regression models do not readily allow for stepwise splits in screening scores which can be accommodated in decision tree analysis. Cross-validated decision trees were constructed with the choice of variables informed by the logistic regression analyses above. A decision tree is a representation of successive splits of the patients into 2 sets according to values of the predictor variables. Here the sets are chosen to maximize the difference in the proportion of patients classified as ‘cases’ between the two sets. The procedure examines every possible split of every predictor variable and chooses the optimal split at each stage. Having held a (random) proportion of patients from the original analysis, the tree is then cross-validated by testing the performance of the resulting classification rule on these excluded patients. This cross-validation procedure avoids over-splitting the dataset, which would result in an overoptimistic decision rule. The decision tree represents a partition of patients into subsets who are more, and less, likely to be classified as ‘cases’. For each tree each ‘terminal node’ contains a proportion of ‘cases’. The threshold proportion for classifying a node as ‘case’ can be varied, resulting in variable values for sensitivity and specificity. This produces a ROC curve for the tree.

The best cross-validated tree informed by Model 2a is shown in Figure 1. As shown the proportions of cases in successive shaded nodes take the values 0, 0.118, 0.329 and 0.812.

If all nodes with proportion of cases > 0.25 are classified as cases a sensitivity of 90% is achieved with 62% specificity. The

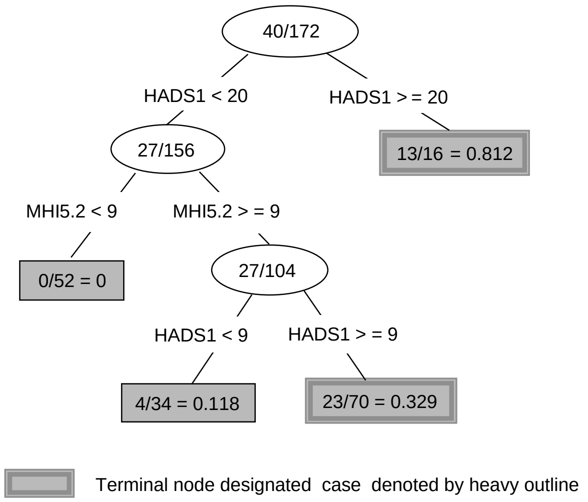

**Figure 1** Decision tree analysis for case-identification using HADS.1 and MH15.2 (Model 2a)

PPV is then 42% and the MR = 31%. The decision rules for screening which emerge from this analysis are thus:

(a) HADS score ≥ 20 = ‘case’
HADS score < 9 = ‘not case’
(b) HADS score ≥ 9, < 20 then administer MHI5
If MHI5 score ≥ 9 = ‘case’; MHI5 score < 9 = ‘not case’

The decision tree analysis for Model 1a highlighted an anomaly in the HADS.2 data: among 19 patients with a score of 12, 13 or 14 there was only 1 ‘case’ as compared with 15 ‘cases’ among the 63 patients scoring ≤ 11. This results in anomalies in the decision tree but does not invalidate the logistic regression model. The latter forces a monotonic relationship which smoothes out irregularities in the data. Data from the first administration of HADS and MHI5 were therefore also explored. Figure 2 shows the best

 *© 2001 Cancer Research Campaign*

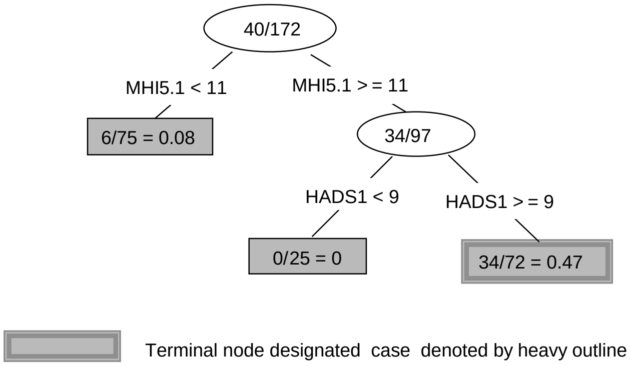

**Figure 2** Decision tree analysis based on MHI5.1 and HADS1

cross-validated tree in which the first node splits patients by MHI5 scores < 11 vs ≥ 11. In the former set, 6 of 75 patients are 'cases' (8%) and this set does not warrant further splits. In the latter set, 34 of 97 patients are 'cases' (35%). In this set a further split based on HADS scores < 9 vs ≥ 9 further improves the classification of 'cases': among those scoring HADS < 9 there are no 'cases' while among those with MHI5 ≥ 11 and HADS ≥ 9 the proportion of cases is now 47% (34 out of 72 patients). If all nodes with proportion of cases > 0.45 are classified as cases (i.e. only the node on the far right of the figure), the decision tree achieves sensitivity of 85% (34/40), specificity of 71% (94/132) and a MR of 26% (6 + 38/172). The PPV is 47%. The decision rules for screening based on this analysis are:

(a) MHI5 score < 11 = 'not case'
(b) MHI5 score ≥ 11 administer HADS
(c) If HADS score < 9 = 'not case'; HADS score ≥ 9 = 'case'.

To test the robustness of this decision tree model these rules were applied to the data derived from HADS and MHI5 at the second assessment. The screening strategy showed a sensitivity of 73% and a specificity of 70% with a misclassification rate of 29% and a positive predictive value of 43%. For ease of comparison the screening properties of the trees are also shown in Table 3.

### DISCUSSION

The aim of our research programme is to develop and test an automated system for collecting and using patient self-report data in clinical practice. The study reported here was a first attempt to harness the potential benefits of the system to the important problem of detecting patients with clinically significant levels of distress.

We selected patients undergoing chemotherapy for our sample for this study. Since they return to hospital on a regular basis they offered us optimal conditions for testing our hypothesis about the value of sequential screening assessments. Specifically we could obtain 2 screening assessments referring to approximately the same time span of the patient's experience as the 'gold standard' interview. We reasoned that if sequential assessment did not improve screening performance in this study it would be unlikely to do so in other areas of oncology practice where the frequency of patient contact is more varied. Within our ongoing programme of work this study was designed to explore the validity of touch-screen data for screening purposes. It was recognized that any screening algorithm developed from this study would require to be validated in an independent sample and that its generalizability would require to be tested in other clinical settings.

The results of computerized screening are encouraging. Patients' scores on questionnaires administered by touchscreen correlated highly with the Catego ID scores derived from their PSE interviews and were predictive of 'caseness'. This supports the validity of using the automated system for collecting patient self-report data about psychological distress. Including sociodemographic data and information about past psychiatric history did not improve the screening performance of the measures selected. Combining data from sequential timepoints did improve the screening performance of the HADS and of the whole assessment battery. However the best screening strategy was derived by combining scores from 2 instruments – MHI5 and HADS, from a single time point. Several issues which limit the interpretation of these findings warrant further discussion.

On a 'gold standard' clinical interview 9% of patients were identified as showing 'case level' symptomatology and a further 14% were 'threshold cases'. It is likely that our sample is biased towards the inclusion of patients who were fitter, physically and/or emotionally. When patients specified reasons for refusal to participate in the study ($n$ = 24/40) the most commonly cited were: 'too distressed' ($n$ = 8); 'can't be bothered' ($n$ = 6) and 'feel too ill' ($n$ = 5). Our previous study (Velikova et al, 1999) found in-patients' refusal to participate related to disease severity. In our on-going work in outpatient clinics (unpublished data), withdrawing from repeated screening is predicted by higher HADS scores at earlier assessment. In the present study data were excluded when patients could not complete the assessments in the time-frame required, i.e. chemotherapy was delayed or discontinued. This usually reflected deterioration in the patient's health. This may well have been associated with distress which we did not capture in this study. For all these reasons it is likely we have reported an underestimate of the prevalence of clinically significant distress in this patient population. Non-compliance with touchscreen assessment in the outpatient setting may in itself be a useful cue to staff to make a clinical assessment of the patients' emotional status. We are evaluating this in our on-going work.

The loss of data for technical reasons was disappointing. There are several possible explanations including: patient error in data entry; error in downloading the data from the touchscreen software to the database within the computer used for data collection; or, error in the subsequent downloading to the computer used for data analysis. It seems unlikely the data were lost by patient error. In most cases the data were lost from only one of 2 assessments and not necessarily the first occasion the patient used the system. Errors in downloading data at the end of clinics would have been expected to affect whole tranches of data rather than the random pattern of loss which we observed. Interview data from patients whose touchscreen data were lost confirms this loss was not a source of systematic bias.

When we became aware of loss of data a number of measures were introduced. Some of the computers used for data-collection were encased to preclude access to the keyboard or barcoders except by members of the team. Data loss was reduced to less than 1% in these computers. The development of this field is not limited by the hardware. New systems, including portable tablet computers and customized computers for touchscreen data collection are now readily available. We have now adopted a rigorous approach to incorporating new hardware and to security issues, which we believe to be essential in this field. The software has

© 2001 Cancer Research Campaign been upgraded to make data entry and download more robust. Training procedures for staff operating the computers in the clinic have been formalized. The problem of data loss has not recurred in our ongoing studies. This reassures us that hardware, software and training solutions are readily available to take forward the reliable use of touchscreens to assist clinic staff in collecting patient self-report data in routine oncology practice.

The patients included in this study were heterogeneous with respect to diagnosis and disease stage, but all were receiving outpatient chemotherapy. Further work is required to validate the use of this screening system among patients with different clinical characteristics. At the time of this study we were not able to derive data electronically from patients records for use in the screening process and could incorporate in the screening algorithm only information which patients could give on arrival at clinic, i.e. sociodemographic details and questionnaire responses, not clinical information. We considered variables known to be associated with increased incidence of psychological morbidity. Age and past psychiatric history were significant predictors of 'caseness', surprisingly, gender was not. When questionnaire scores were included in the predictive model there was no evidence that any of these variables contributed to the screening performance of the instruments used in this study.

Patients' questionnaire scores on screening were not significantly different across the 2 assessments. The measures used were significantly correlated with each other and individually, with Catego ID scores. Taken singly, each was a highly significant predictor of 'caseness'. The MHI5 and HADS were the best single predictors of 'caseness'. As these were designed as screening measures this is not surprising. Its short time-frame relative to that used in standard psychiatric interviews, may compromise the screening performance of the HADS. Our results confirmed that using sequential HADS scores did significantly improve screening performance over a single HADS. Including all questionnaire data from sequential assessments, 2–4 weeks apart, improved the regression model.

This preliminary study did not have the power to detect statistically significant differences between all the possible alternative models for combining data from the 2 assessments. Comparison of regression models suggested an advantage to combining scores from MHI5 and HADS to predict 'caseness'. Replication in a much larger sample would be needed to confirm the optimal use of these instruments. The screening performance of the best regression model (i.e. Model 2a, HADS.1 + MHI5.2) was disappointing (Table 3). We therefore considered a non-linear solution. Decision tree analysis allows stepwise splits in the screening scores. The screening rules derived from this analysis (Figure 1) allowed Catego 'cases' to be identified with a high level of sensitivity (90%) but at a cost of a rather low specificity (62%). This model was illogical clinically, presenting the longer instrument before the shorter and potentially better predictor. We therefore explored other solutions. The decision-tree method was also applied to Model 1a (HADS.2 + MHI5.2). With some splits in the data a higher proportion of 'cases' was revealed among patients with lower vs higher HADS scores. These chance findings did not invalidate the linear regression analysis but decision analysis based on those thresholds would be clinically meaningless. Technically the best screening strategy, identified by the tree method (Figure 2 – MHI5.1 + HADS.1), is satisfying both for its clinical logic and screening performance. The data used are derived from a single assessment and screening begins with the shorter instrument – MHI5. Only patients who score > 11 need be asked to complete the HADS. This strategy correctly identified 85% of Catego 'cases'. Of the 6 'cases' misclassified as 'not cases', two thirds had a Catego score of 5, i.e. 'threshold disorder'. The specificity and positive predictive value of this strategy were somewhat better than for the tree based on Model 2a, implying greater efficiency in the use of staff time to assess screen-positive patients. Replication in another sample of patients drawn from the same population would be required before this preferred screening strategy could be recommended.

In busy clinical practice it is important to weigh the benefits of maximizing 'case-identification' against the cost of flagging a high number of 'non-cases' to staff as requiring fuller assessment. In our study a moderate false-negative rate was acceptable because patients were returning to hospital and interacting with health professionals on a regular basis, giving other opportunities for 'cases' missed on screening to be picked up subsequently. It is important to stress that however effective the screening system eventually developed, it is intended as an aid to, and not a substitute for, doctor–patient communication. We have also suggested that patients' non-participation in screening may be informative as a cue to clinical assessment.

There is clearly scope for using the touchscreen system to explore the screening performance of other combinations of items and scales. Recently the GHQ-12 has been recommended as an alternative to the HADS (Hall et al, 1999) in oncology clinics. Single items such as – 'Are you depressed?' – which has been found reliable and valid as a screening tool among terminally ill patients (Chochinov et al, 1997), also warrant further consideration here. Most diagnostic interviews have a structure in which response to an opening question determines whether more detailed enquiry about psychological symptoms is pursued. A similar branching approach in computerized screening may be useful and should be further explored.

Health-related quality of life (QL) assessment for the future is moving towards computer-adaptive testing (Revicki and Cella, 1997) based on the application of item response theory (IRT) to characterize whole banks of questionnaire items for a number of QL dimensions. This approach, first used in educational assessment, holds the promise of enabling the generation of brief and more precise assessments of psychological morbidity tailored to the individual patient. Data collection by touchscreen computer in routine oncology practice offers the opportunity to improve the identification of patients with clinically significant levels of distress. A recent systematic review (Gilbody et al, 2001) examining evidence of the effect of routine screening on the recognition, management and outcome of psychiatric disorders in non-psychiatric settings, confirmed the value of providing clinicians with feedback only about those who screen positive. Computer-administered screening is a promising platform from which to develop a rational strategy for offering a range of psychosocial interventions appropriately to patients most in need of them. Further work is needed to develop the optimal selection of screening questions for this purpose.

### ACKNOWLEDGEMENTS

This research was supported by grants from the NHS National Research and Development Programme, the Imperial Cancer Research Fund (AC, GV, PS) and the National Lotteries Charities Board (GV). Carstairs scores were provided by the Office of National Statistics, UK. The software was designed by AS who

*© 2001 Cancer Research Campaign*

can be contacted for further information at the ICRF Cancer Medicine Research Unit, Leeds, UK.

### REFERENCES

Aapro M and Cull A (1999) Depression in breast cancer patients: the need for treatment. *Annal Oncol* **10**: 627–636

Aaronson NK, Ahmedzai S, Bergman B et al (1993) The European Organization for Research and Treatment of Cancer (EORTC): A quality of life instrument for use in international clinical trials in oncology. *J Natl Cancer Inst* **85**: 365–376

Armitage P (1971) *Statistical Methods in Medical Research*. Blackwell: Oxford

Berard RMF, Boer Meester F and Viljoen G (1998) Depressive disorders in an outpatient oncology Setting: Prevalence Assessment and Management. *PsychoOncology* **7**: 112, 120

Berwick DM, Murphy JM, Goldman PA et al (1991) Performance of a five item mental health screening test. *Medical Care* **29**: 169–176

Carstairs V and Morris R (1991) *Deprivation and Health in Scotland*. Aberdeen University Press: Aberdeen

Chochinov HM, Wilson KG, Enns M et al (1997) Are you depressed? Screening for depression in the terminally ill. *Am J Psychiatry* **154**: 674–676

Cull A, Stewart M and Altman DG (1995) Assessment of and Intervention for Psychosocial Problems in Routine Oncology Practice. *Brit J Cancer* **72**: 229–235

Detmar SB and Aaronson NK (1998) Quality of Life Assessment in Daily Clinical Oncology Practice: A feasibility study. *Eur J Cancer* **34**: 8 1181–1186

Fallowfield L, Lipkin M and Hall A (1998) Helping senior oncologists communication skills: Results from Phase 1 of a Comprehensive Longitudinal Program in the United Kingdom. *J Clin Oncol*. **16**(**5**): 1961–1968

Fallowfield L, Ratcliffe D, Jenkins V et al (2001) Psychiatric morbidity and its recognition by doctors in patients with cancer. *Brit J Cancer* 84(8) 1011–1015

Gilbody SM, House AO and Sheldon TA (2001) Routinely administered questionnaires for depression and anxiety: systematic review. *Brit Med J* **322**: 406–409

Hall A, A'Hern R and Fallowfield L (1999) Are we using appropriate self report questionnaires for detecting anxiety and depression in women with early breast cancer. *Eur J Cancer* **35** (**1**): 79–85

Harrell FE, Margolis PA, Gove S et al (1998) Development of a clinical prediction model for ordinal outcomes. *Statistics in Medicine* **17**: 909–944

Harrell FE (2000). *Regression Modeling Strategies with Applications to Survival Analysis* and *Logistic Regression*. Springer-Verlag: New York

Harrison J, Maguire P, Ibbotson T et al (1994) Concerns cofiding and psychiatric disorder in newly diagnosed cancer patients: A descriptive study. *PsychoOncology* **3**: 173–179

Hopwood P, Howell A and Maguire P (1991a). Psychiatric morbidity in patients with advanced cancer of the breast: prevalence by two self rating questionnaires. *Brit J Cancer* **64**: 349–352

Hopwood P, Howell A and Maguire P (1991b) Screening for psychiatric morbidity in patients with advanced breast cancer: validation of two self report questionnaires. *Brit J Cancer* **64**: 353–356

Ibbotson T, Maguire P, Selby P et al (1994) Screening for Anxiety and Depression in Cancer Patients: The effects of disease and treatment. *Eur J Cancer* **30A**(**1**): 37–40

Maguire P (1985) Psychological morbidity associated with cancer and cancer treatment. *Clin Oncol* **4**: 559–575

Maguire P, Faulkner A, Booth K et al (1996) Helping cancer patients disclose their concerns. *Eur J Cancer* **32A**: 78–81

McCullagh P and Nelder JA(1989) Generalised Liner Models. Chapman and Hall: London

Meyer TJ and Mark MM (1995) Effects of psychosocial interventions with adult cancer patients: A meta analysis of randomised experiments. *Health Psychology* **14**: 101–108

Murphy J, Berwick D, Weinstein M et al (1987) Performance of screening and diagnostic tests: application of receiver operating characteristic analysis. *Arch Gen Psychiatr* **44**: 550–555

Newell S, Samson-Fisher R and Girgis A (1998) How well do medical oncologists perceptions reflect their patients reported physical and psychosocial problems? Cancer **83** (**8**): 1640–1651

Pinder KL, Ramirez AJ, Black E et al (1993) Psychiatric disorders in patients with advanced breast cancer: prevalence and associated factors. *Eur J Cancer* **29A**(**4**): 524–527

Ramirez AJ and House A (1997) Common mental health problems in hospital. *Brit Med J* **314**: 1679–1681

Ramirez AJ, Richards MA, Jarrett SR et al (1995) Can mood disorder in women with breast cancer be identified pre-operatively? *Brit J Cancer* **72**: 1509–1512

Razavi D, Delvaux N, Faracques C et al (1990) Screening for adjustment disorders and major depressive disorders in cancer in patients. *Brit J Psychiatry* **156**: 79–83

Razavi D, Delvaux N, Bredart A et al (1992) Screening for psychiatric disorders in a lymphoma outpatient population. *Eur J Cancer* **28A**(**ii**): 1869–1872

Revicki DA and Cella DF (1997) Health status assessment for the twenty-first century: Item response theory, item banking and computer adaptive testing. *Quality of Life Research* **6**: 595–600

Robertson MM and Katona CC (1997) Depression and Physical Illness. John Wiley and Sons: Chichester

S-Plus User's Guide (1997) Data Analysis Products Division, Mathsoft: Seattle

Velikova G, Wright EP, Smith et al (1999) Automated collection of quality of life data: A comparison of paper and computer touchscreen questionnaires. *J Clin Oncol* **17**: 998–1007

Venables WN and Ripley BD (1997) *Modern Applied Statistics with S-Plus*. Springer: New York

Ware J E (1993) SF-36 *Health Survey: Manual and Interpretation Guide*. New England Medical Center: Boston, MA

Wing J, Cooper J and Sartorious N (1974) *Measurement and Classification of Psychiatric Symptoms*. Cambridge: Cambridge University Press

Zigmond A and Snaith R (1983) The Hospital Anxiety and Depression Scale. *Acta Psychiatr Scand*. **67**: 361–367

*© 2001 Cancer Research Campaign*
